# A Study of Intradialytic Complications in Chronic Kidney Disease Patients

**DOI:** 10.7759/cureus.105218

**Published:** 2026-03-14

**Authors:** Basavaraj Nilugal, Siddanagouda M Biradar, Sandeep Patil

**Affiliations:** 1 General Medicine, Shri BM Patil Medical College, Hospital and Research Centre, BLDE (Deemed to be University), Vijayapura, IND

**Keywords:** chronic kidney disease, hemodialysis, hypotension, intradialytic complications, ultrafiltration

## Abstract

Introduction: Chronic kidney disease (CKD) is a significant public health issue worldwide, with the need for renal replacement therapy at advanced stages. Hemodialysis is a life-sustaining treatment; however, several intradialytic complications occur during the treatment. The complications may impact the safety and comfort of the patient and the adequacy of the treatment. The present study aimed to determine the incidence and pattern of intradialytic complications and to evaluate clinical factors associated with their occurrence among chronic kidney disease patients undergoing hemodialysis.

Materials and methods: An observational cross-sectional study was conducted at a tertiary care hospital in Vijayapura, Karnataka, India, from June 2024 to December 2025. A total of 90 CKD patients undergoing maintenance hemodialysis were included, and 205 hemodialysis sessions were prospectively observed. Intradialytic complications were identified using standard clinical definitions. Blood pressure and clinical parameters were recorded pre-dialysis, at hourly intervals during dialysis, and immediately post-dialysis. Data were analyzed using IBM SPSS Statistics, version 26 (IBM Corp., Armonk, NY, USA) and presented as mean ± standard deviation or frequency and percentage.

Results: A total of 205 hemodialysis sessions were analyzed, of which 68 (33.2%) were complicated. Intradialytic hypotension was the most frequent complication, occurring in 42 (20.4%) sessions. Intradialytic hypertension occurred in 16 (23.5%) complicated sessions. Sessions complicated by hypotension were associated with higher ultrafiltration goal, higher ultrafiltration rate, higher pre-dialysis systolic blood pressure, and greater interdialytic weight gain compared with non-hypotensive sessions.

Conclusion: Intradialytic complications occurred in a considerable proportion of hemodialysis sessions, with hypotension being the most common event. Higher ultrafiltration rates and greater interdialytic weight gain were associated with hypotensive episodes. Careful monitoring of hemodynamic parameters and optimization of fluid removal strategies are essential to improve patient safety during hemodialysis.

## Introduction

Chronic kidney disease (CKD) is a major global public health problem and a significant contributor to morbidity, mortality, and healthcare expenditure worldwide [1]. It is estimated that nearly 850 million people are affected by various forms of kidney disease globally [2]. As the disease progresses to end-stage renal disease (ESRD), renal replacement therapy becomes essential for survival, and the most commonly used modality is hemodialysis [3]. Despite improvements in dialysis machines and dialysis techniques, hemodialysis is associated with several intradialytic complications, which pose important challenges in the management of patients undergoing dialysis [4]. These complications affect patient comfort, treatment adequacy, quality of life, and overall healthcare costs [5]. Hence, understanding the incidence, pattern, and contributing factors of intradialytic complications is of considerable clinical interest [3].

Hemodialysis is a physiologically stressful procedure that involves rapid shifts in blood volume, electrolytes, osmotic balance, and acid-base status over a relatively short period of time [6]. These rapid physiological changes are known to contribute to various complications, among which intradialytic hypotension is the most frequently reported event during hemodialysis sessions [7]. The mechanism underlying intradialytic hypotension is complex and may involve excessive ultrafiltration, impaired cardiovascular compensatory responses, autonomic dysfunction, and reduced plasma refilling capacity [8]. This condition may adversely affect vital organs such as the myocardium and brain, potentially leading to cellular injury and worsening clinical outcomes [9]. Another common complication is muscle cramps, which are involuntary and painful contractions usually occurring during the latter half of the dialysis session and are frequently related to aggressive fluid removal and attempts to achieve dry weight [10]. Severe muscle cramps may lead to premature termination of dialysis sessions, resulting in inadequate dialysis delivery and increased morbidity [10].

Other important intradialytic complications include disequilibrium syndrome, arrhythmias, chest pain, and hypersensitivity reactions [11]. Although dialysis disequilibrium syndrome (DDS) has become less common with advances in dialysis technology, it may still occur in newly initiated dialysis patients with severe uremia due to osmotic shifts and cerebral edema [12]. Cardiovascular complications are also a major concern in the end-stage renal disease (ESRD) population, as rapid electrolyte fluctuations during dialysis may predispose patients to arrhythmias and sudden cardiac death [13]. Hypersensitivity reactions to dialysis membranes or medications may range from mild cutaneous manifestations to severe anaphylactic reactions [7,11]. In addition, the occurrence of intradialytic complications may have significant psychological consequences, including anxiety and depression among patients undergoing long-term hemodialysis [14].

Given the wide range of physiological disturbances associated with hemodialysis, systematic evaluation of intradialytic complications and their associated clinical parameters is important for improving patient safety and optimizing dialysis management. Therefore, the present study aimed to determine the incidence and pattern of intradialytic complications and to evaluate selected clinical factors associated with their occurrence among CKD patients undergoing hemodialysis.

## Materials and methods

This observational cross-sectional study was conducted in the Department of General Medicine at Shri B. M. Patil Medical College, Hospital and Research Centre, Vijayapura, Karnataka, India, over a period of 18 months from June 2024 to December 2025. The Institutional Ethics Committee approval number was BLDE(DU)/IEC-SBMPMC/040/2023-24.

The formula used to calculate the sample size was

$n = \frac{Z^2 \times p(1 - p)}{d^2}$,

where Z = 1.96 at 95% confidence interval, p = 0.307 (30.7%) as per the previously reported prevalence of intradialytic complications in patients undergoing hemodialysis [7], and d = 0.096 (9.6% absolute precision). Hence, the calculated minimum required sample size was 88. During the study period of 18 months, 90 adult patients with CKD on maintenance hemodialysis and a total of 205 hemodialysis sessions were included to assess intradialytic complications.

The study included all patients aged over 18 years with established CKD on maintenance hemodialysis. CKD was defined as structural and/or functional abnormalities of the kidney persisting for over three months, with or without decreased glomerular filtration rate [15]. Patients who were pregnant or who had dementia/disorientation and were unable to provide a reliable clinical history were excluded from the study. Both inpatients and outpatients on hemodialysis during the study period were included. Written informed consent was obtained from all participants.

A detailed clinical history was obtained, including age, sex, duration of chronic kidney disease, duration and frequency of hemodialysis (once, twice, or thrice weekly), and the presence of any comorbid conditions. Patients were receiving dialysis according to the schedule prescribed by the treating nephrologist based on clinical status and resource availability. Physical examination was performed, including height, weight, pulse rate, and blood pressure.

Systolic blood pressure (SBP) and diastolic blood pressure (DBP) were recorded using a calibrated sphygmomanometer before initiation of dialysis, at hourly intervals during the dialysis session, and immediately after completion of dialysis. The mean arterial pressure (MAP) was calculated using the formula: 

$\mathrm{MAP} = \mathrm{DBP} + \frac{\mathrm{SBP} - \mathrm{DBP}}{3}$.

The interdialytic weight gain (IDWG) was defined as the difference between the pre-dialysis weight of the current session and the post-dialysis weight of the previous session, while post-dialytic weight loss was defined as the difference between pre-dialysis and post-dialysis weights of the same session [16].

The ultrafiltration (UF) goal was defined as the total volume of fluid to be removed during a dialysis session, expressed in liters, while the ultrafiltration rate (UFR) was calculated as total ultrafiltration volume divided by dialysis duration and body weight, with a target value of less than 10 mL/kg/hour for all patients [17]. The laboratory parameters studied included complete blood count, random blood glucose, serum electrolytes, creatinine, blood urea, and uric acid levels.

Vascular access was classified as an arteriovenous fistula, an internal jugular venous catheter, and a femoral venous catheter. Each session of hemodialysis was continuously monitored by trained dialysis staff for the occurrence of intradialytic complications through clinical observation and bedside monitoring.

Intradialytic hypotension was defined as a fall in systolic blood pressure of ≥20 mmHg from baseline or a systolic blood pressure <90 mmHg during dialysis, with or without associated symptoms [7]. Intradialytic hypertension was defined as an elevation in mean arterial pressure by >15 mmHg during or immediately after dialysis [7]. Hypoglycemia was defined as blood glucose levels <70 mg/dL with or without symptoms [18]. Fever was defined as a body temperature ≥38°C during dialysis [19]. Chills, nausea, vomiting, muscle cramps, headache, chest pain, and back pain were identified based on patient-reported symptoms during dialysis and clinical assessment by the attending dialysis staff. Arrhythmias were detected using continuous cardiac monitoring available on the dialysis machine, while disequilibrium syndrome was identified based on neurological symptoms such as headache, nausea, restlessness, or altered sensorium occurring during dialysis. Access-related complications, including bleeding, catheter malfunction, or access-site infection, were recorded based on clinical examination and dialysis unit records if they occurred during the hemodialysis session.

Data were entered into Microsoft Excel (Microsoft Corp., Redmond, WA, USA) and analyzed using IBM SPSS Statistics, version 26 (IBM Corp., Armonk, NY, USA). Quantitative variables were presented as mean ± standard deviation, while categorical variables were presented as frequency and percentage. Comparisons between hypotensive and non-hypotensive sessions were performed using the independent samples t-test. A p-value <0.05 was considered statistically significant.

## Results

The study included 90 patients undergoing maintenance hemodialysis during the study period. Male patients constituted 63 (70.0%) of the study population, while female patients accounted for 27 (30.0%). Regarding dialysis frequency, the majority of patients were receiving dialysis twice weekly at 54 (60.0%), followed by thrice weekly dialysis at 21 (23.3%), and once weekly dialysis at 15 (16.7%) (Table 1).

**Table 1:** Baseline characteristics of patients (N = 90) Data are presented as number and percentage (n (%)).

Variable	Category	n (%)
Sex	Male	63 (70.0%)
	Female	27 (30.0%)
Dialysis frequency/week	Once	15 (16.7%)
	Twice	54 (60.0%)
	Thrice	21 (23.3%)

With respect to comorbid conditions, the most common category was combined hypertension and type 2 diabetes mellitus, observed in 38 (42.2%) patients. Isolated hypertension was present in 35 (38.8%), while isolated type 2 diabetes mellitus was seen in three (3.3%) patients. One patient (1.1%) had ischemic heart disease along with hypertension and diabetes. Dilated cardiomyopathy was noted in two (2.2%) patients, and renal structural defects were present in three (3.3%). A total of eight (8.8%) patients had no documented comorbidities (Table 2).

**Table 2:** Comorbidities among patients (N = 90) Data are expressed as number and percentage (n (%)). HTN: hypertension; T2DM: type 2 diabetes mellitus; IHD: ischemic heart disease

Comorbidity	n (%)
Hypertension (HTN)	35 (38.8%)
Type 2 diabetes mellitus (T2DM)	3 (3.3%)
HTN + T2DM	38 (42.2%)
IHD + T2DM + HTN	1 (1.1%)
Dilated cardiomyopathy	2 (2.2%)
Renal structural defect	3 (3.3%)
No comorbidities	8 (8.8%)

A total of 205 hemodialysis sessions were analyzed at the session level. Arteriovenous fistula was the most frequently used vascular access, accounting for 142 sessions (69.3%). Jugular catheter access was used in 59 sessions (28.8%), while femoral catheter access was the least common, used in four sessions (2.0%) (Table 3).

**Table 3:** Distribution of vascular access across hemodialysis sessions (n = 205) Data are presented as number and percentage (n (%)).

Vascular access	n (%)
Arteriovenous fistula	142 (69.3%)
Jugular catheter	59 (28.8%)
Femoral catheter	4 (2.0%)

Among the 205 hemodialysis sessions analyzed, 68 sessions (33.2%) were complicated by at least one intradialytic event, whereas 137 sessions (66.8%) were uneventful (Table 4).

**Table 4:** Frequency of intradialytic complications (session-level) (n = 205) Data are presented as number and percentage (n (%)).

Session outcome	n (%)
Complicated sessions	68 (33.2%)
Uncomplicated sessions	137 (66.8%)

Among the 68 complicated sessions, hypotension was the most frequent complication, occurring in 42 sessions (61.8%). Hypertension was observed in 16 sessions (23.5%). Other less frequent complications included chills at two (2.9%) sessions, nausea/vomiting at two (2.9%), hypoglycemia at two (2.9%), pain or muscle cramps at two (2.9%), hypoglycemia with giddiness at one (1.4%), and fever with chills at one (1.4%) (Table 5).

**Table 5:** Types of intradialytic complications (among complicated sessions, n = 68) Data are presented as number and percentage (n (%)) of complicated sessions.

Complication	n (%)
Hypotension	42 (61.8%)
Hypertension	16 (23.5%)
Fever with chills	1 (1.4%)
Chills	2 (2.9%)
Nausea/vomiting	2 (2.9%)
Hypoglycemia	2 (2.9%)
Hypoglycemia with giddiness	1 (1.4%)
Pain/muscle cramps	2 (2.9%)

When complicated sessions were analyzed according to comorbidity status, hypertension alone was associated with 30 sessions (44.1%), while combined hypertension and type 2 diabetes mellitus accounted for 25 sessions (36.8%). Type 2 diabetes mellitus alone was present in three sessions (4.4%). Dilated cardiomyopathy and renal structural defects each accounted for two sessions (2.9%). A total of six sessions (8.8%) occurred in patients without documented comorbidities (Table 6).

**Table 6:** Complicated sessions according to comorbidity (session-level, n = 68) Data are expressed as number and percentage (n (%)) of total complicated sessions. HTN: hypertension; T2DM: type 2 diabetes mellitus

Comorbidity	n (%)
Hypertension	30 (44.1%)
T2DM	3 (4.4%)
HTN + T2DM	25 (36.8%)
Dilated cardiomyopathy	2 (2.9%)
Renal structural defect	2 (2.9%)
No comorbidities	6 (8.8%)

Analysis of complicated sessions according to vascular access revealed that 44 sessions (64.7%) occurred with an arteriovenous fistula, 23 sessions (33.8%) with a jugular catheter, and one session (1.4%) with a femoral catheter (Table 7).

**Table 7:** Complicated sessions according to vascular access (n = 68) Data are presented as number and percentage (n (%)) of complicated sessions.

Vascular access	n (%)
Arteriovenous fistula	44 (64.7%)
Jugular catheter	23 (33.8%)
Femoral catheter	1 (1.4%)

When complications were distributed according to dialysis frequency, hypotension was most commonly observed in twice-weekly dialysis sessions (30 sessions, 44.1%), followed by thrice-weekly sessions (eight sessions, 11.7%) and once-weekly sessions (four sessions, 5.8%). Hypertension was distributed as seven (10.3%) in twice-weekly sessions, five (7.3%) in thrice-weekly sessions, and four (5.8%) in once-weekly sessions (Table 8).

**Table 8:** Distribution of complications according to dialysis frequency (n = 68) Data are expressed as number and percentage (n (%)) of total complicated sessions across dialysis frequency categories.

Complication	Once/week n (%)	Twice/week n (%)	Thrice/week n (%)	Total n (%)
Hypotension	4 (5.8%)	30 (44.1%)	8 (11.7%)	42 (61.8%)
Hypertension	4 (5.8%)	7 (10.3%)	5 (7.3%)	16 (23.5%)
Fever with chills	0 (0%)	1 (1.4%)	0 (0%)	1 (1.4%)
Chills	0 (0%)	2 (2.9%)	0 (0%)	2 (2.9%)
Nausea/vomiting	1 (1.4%)	1 (1.4%)	0 (0%)	2 (2.9%)
Hypoglycemia	0 (0%)	2 (2.9%)	0 (0%)	2 (2.9%)
Hypoglycemia + giddiness	0 (0%)	0 (0%)	1 (1.4%)	1 (1.4%)
Pain/cramps	0 (0%)	1 (1.4%)	1 (1.4%)	2 (2.9%)

Comparison between hypotensive and non-hypotensive sessions demonstrated that hypotensive sessions had higher mean ultrafiltration goal (1.94 ± 0.51 L vs 1.29 ± 0.42 L), higher ultrafiltration rate (0.58 ± 0.14 L/hr vs 0.37 ± 0.11 L/hr), higher pre-dialysis systolic blood pressure (159.4 ± 21.3 mmHg vs 146.9 ± 18.6 mmHg), and greater interdialytic weight gain (1.70 ± 0.45 kg vs 1.23 ± 0.38 kg). Post-dialysis systolic blood pressure was lower in hypotensive sessions (138.6 ± 19.5 mmHg) compared to non-hypotensive sessions (153.1 ± 17.8 mmHg). Dialysis duration was comparable between groups (3.85 ± 0.36 hours vs 3.75 ± 0.32 hours) (Table 9).

**Table 9:** Comparison of hypotensive vs. non-hypotensive sessions (n = 205) Data are presented as mean ± standard deviation (SD). Comparison between groups was performed using the independent samples t-test. UF: ultrafiltration; SBP: systolic blood pressure; DBP: diastolic blood pressure

Variable	Non-hypotensive sessions (n = 163)	Hypotensive sessions (n = 42)	t value	p value
UF goal (L)	1.29 ± 0.42	1.94 ± 0.51	-7.62	<0.001
UF rate (L/hr)	0.37 ± 0.11	0.58 ± 0.14	-9.03	<0.001
Pre-dialysis SBP (mmHg)	146.9 ± 18.6	159.4 ± 21.3	-3.48	<0.001
Pre-dialysis DBP (mmHg)	81.6 ± 10.4	87.4 ± 12.1	-2.85	0.004
Post-dialysis SBP (mmHg)	153.1 ± 17.8	138.6 ± 19.5	4.37	<0.001
Post-dialysis DBP (mmHg)	84.8 ± 9.6	82.1 ± 10.3	1.54	0.125
Inter-dialytic weight gain (kg)	1.23 ± 0.38	1.70 ± 0.45	-6.22	<0.001
Post-dialytic weight loss (kg)	1.11 ± 0.35	1.60 ± 0.41	-7.11	<0.001
Duration (hours)	3.75 ± 0.32	3.85 ± 0.36	-1.64	0.101

## Discussion

The present study found a male predominance among patients undergoing maintenance hemodialysis. There were 63 (70%) male and 27 (30%) female patients. This finding is similar to the previously published literature showing a higher prevalence of CKD and ESRD in males. Narayanaswamy et al. found a similar male predominance in their study from Karnataka [20]. Similarly, Dugilo et al. reported comparable findings in their study from Tanzania [21]. The increased representation of males may reflect a higher burden of hypertension, diabetes, and cardiovascular risk factors, as well as differences in healthcare access and health-seeking behaviour.

Twice-weekly hemodialysis was found to be the most common schedule among the patients. This was followed by thrice-weekly and once-weekly hemodialysis. There were 54 (60%) patients on twice-weekly hemodialysis. This was followed by 21 (23.3%) patients on thrice-weekly hemodialysis and 15 (16.7%) patients on once-weekly hemodialysis. This finding is similar to the previously published literature. Raja & Seyoum found no significant difference in complication rates between twice-weekly and thrice-weekly hemodialysis [7]. However, proper fluid management was emphasized. Bartaula et al. found irregular dialysis schedules to be associated with higher complication rates (p < 0.001) [22]. These observations suggest that, regardless of dialysis frequency, careful monitoring of fluid removal and hemodynamic parameters remains essential to minimize intradialytic complications.

The predominant comorbidity profile in our cohort was combined hypertension and diabetes in 38 (42.2%) patients, followed by hypertension alone in 35 (38.8%). This aligns with global epidemiological trends where diabetes and hypertension are leading causes of ESRD. Narayanaswamy et al. reported high rates of hypertension and diabetes, while Sah et al. identified strong associations between diabetes, hypertension, and complications such as arrhythmia and glycemic disturbances [20,23]. Patil et al. emphasized that concomitant hypertension and diabetes exacerbate dialysis-related complications and recommended early risk identification [24]. Among 42 hypotensive sessions in our study, 22 (52.3%) occurred in hypertensive patients and 17 (40.4%) in patients with both hypertension and diabetes, underscoring the vulnerability of this comorbid phenotype. These findings indicate that patients with cardiovascular and metabolic comorbidities may have reduced hemodynamic reserve during dialysis, predisposing them to intradialytic blood pressure fluctuations.

Arteriovenous fistula was the most commonly used vascular access, accounting for 57 (63.3%) patients and 142 (69.3%) of 205 sessions. Jugular catheters accounted for 59 (28.8%) sessions, and femoral catheters accounted for four (2%) sessions. Among 68 complicated sessions, 44 (64.7%) occurred in fistula-based sessions and 23 (33.8%) in jugular catheter sessions. Although central venous catheters are traditionally associated with higher infection and complication risks, as noted by Raja & Seyoum, the higher absolute number of complications in fistula sessions in our study likely reflects their greater overall utilization rather than intrinsic risk [7]. This observation highlights that the distribution of complications across vascular access types should be interpreted in the context of their frequency of use rather than as a direct indicator of access-related risk.

The overall percentage of intradialytic complications in the present study was 68 (33.2%) out of 205 sessions. This percentage is comparable to that found in the study by Raja & Seyoum (30.7%) but lower than that reported in studies by Narayanaswamy et al. (84.2%) and Fatima et al. (86.6%) [7,20,25]. Hypotension was the most common complication in the present study, accounting for 42 (20.4%) of all sessions and 42 (61.8%) of 68 complicated sessions. Similar results were also found in studies by Hasan et al. (26.8%) and Patil et al. (34.2%) [24,26]. Hypertension (16; 23.5%), chills (2; 2.9%), nausea/vomiting (2; 2.9%), hypoglycemia (2; 2.9%), and cramps (2; 2.9%) were less common in the present study compared to other studies conducted in different countries. Dugilo et al. also reported higher percentages of intradialytic hypertension in their study [21]. These differences could be due to regional variations in patient characteristics, dialysis practices, and fluid management strategies. Furthermore, in the present study, hypotensive sessions were associated with higher ultrafiltration goals, higher ultrafiltration rates, and greater interdialytic weight gain, suggesting that aggressive fluid removal may contribute significantly to hemodynamic instability during dialysis.

Effective management of intradialytic hypotension primarily focuses on preventive strategies and optimization of fluid removal during hemodialysis. Measures such as reducing ultrafiltration rates, careful assessment of dry weight, adjusting dialysate temperature, and close hemodynamic monitoring may help improve cardiovascular stability during dialysis sessions. In addition, pharmacological approaches may be considered in selected patients with recurrent intradialytic hypotension. A randomized controlled trial by Ibarra-Sifuentes et al. demonstrated that levocarnitine supplementation administered before hemodialysis sessions significantly reduced the frequency of intradialytic hypotension episodes, suggesting a potential therapeutic option for patients with recurrent episodes [27].

The present study has certain limitations that must be acknowledged. The study was conducted at a single centre with a relatively small sample size of 90 patients. The cross-sectional design precludes assessment of long-term outcomes and causal relationships. Additionally, clustering of multiple sessions within the same patient was not adjusted for in statistical analysis, which may influence the precision of estimates. Furthermore, detailed baseline laboratory parameters, hemodialysis vintage, dry weight, and medication use were not systematically recorded in the study dataset, which limited the ability to analyze their potential association with intradialytic complications. Therefore, the findings should be interpreted with caution when extrapolating to other dialysis populations or healthcare settings. Despite these limitations, the study provides valuable insight into the burden and pattern of intradialytic complications in our setting.

## Conclusions

Intradialytic complications were observed in a substantial proportion of hemodialysis sessions among patients with chronic kidney disease. Hypotension was found to be the most common intradialytic complication, accounting for the majority of complicated dialysis sessions in the present study. Other intradialytic complications, including intradialytic hypertension, chills, hypoglycemia, nausea, and muscle cramps, were observed less frequently. Sessions complicated by hypotension were associated with higher ultrafiltration goals, higher ultrafiltration rates, and greater interdialytic weight gain, suggesting that fluid removal dynamics play an important role in the development of intradialytic instability. This study emphasizes the importance of careful hemodynamic monitoring and individualized fluid management during hemodialysis sessions to minimize complications and improve patient safety. The findings provide insight into the pattern of intradialytic complications in our clinical setting and highlight the need for continuous surveillance and optimization of dialysis practices in patients undergoing maintenance hemodialysis.

## References

[1] Xie K, Cao H, Ling S, Zhong J, Chen H, Chen P, Huang R (2025). Global, regional, and national burden of chronic kidney disease, 1990-2021: a systematic analysis for the global burden of disease study 2021. Front Endocrinol (Lausanne).

[2] Li PK, Chan GC, Chen J (2021). Tackling dialysis burden around the world: a global challenge. Kidney Dis (Basel).

[3] Subhan A, Siddharth L, Saba S, Khanam S, Goud MR (2025). An insight to kidney dialysis treatment. Int J Basic Clin Pharmacol.

[4] Haddiya I, Simanjuntak GD, Ramdani S (2025). Updates in the management of intradialytic hypotension: emerging strategies and innovations. World J Nephrol.

[5] Flythe JE, Xue H, Lynch KE, Curhan GC, Brunelli SM (2015). Association of mortality risk with various definitions of intradialytic hypotension. J Am Soc Nephrol.

[6] Canaud B, Chazot C, Koomans J, Collins A (2019). Fluid and hemodynamic management in hemodialysis patients: challenges and opportunities. J Bras Nefrol.

[7] Raja SM, Seyoum Y (2020). Intradialytic complications among patients on twice-weekly maintenance hemodialysis: an experience from a hemodialysis center in Eritrea. BMC Nephrol.

[8] Sars B, van der Sande FM, Kooman JP (2020). Intradialytic hypotension: mechanisms and outcome. Blood Purif.

[9] Dasgupta I, Thomas GN, Clarke J (2019). Associations between hemodialysis facility practices to manage fluid volume and intradialytic hypotension and patient outcomes. Clin J Am Soc Nephrol.

[10] Kot G, Wróbel A, Kuna K, Makówka A, Nowicki M (2024). The effect of muscle cramps during hemodialysis on quality of life and habitual physical activity. Medicina (Kaunas).

[11] Singh P, Gopal R, Singh S, Rathore SS, Choudhary TA (2015). Spectrum of intradialytic complications during hemodialysis and its management: a single-center experience. Saudi J Kidney Dis Transpl.

[12] Mistry K (2019). Dialysis disequilibrium syndrome prevention and management. Int J Nephrol Renovasc Dis.

[13] Makar MS, Pun PH (2017). Sudden cardiac death among hemodialysis patients. Am J Kidney Dis.

[14] Gerogianni G, Polikandrioti M, Babatsikou F (2019). Anxiety-depression of dialysis patients and their caregivers. Medicina (Kaunas).

[15] (2013). Chapter 1: Definition and classification of CKD. Kidney Int Suppl (2011).

[16] Jalalzadeh M, Mousavinasab S, Villavicencio C, Aameish M, Chaudhari S, Baumstein D (2021). Consequences of interdialytic weight gain among hemodialysis patients. Cureus.

[17] Imaizumi T, Okazaki M, Hishida M (2025). Ultrafiltration rate and mortality in patients undergoing extended-hours haemodialysis. Clin Kidney J.

[18] Kang DH, Streja E, You AS (2024). Hypoglycemia and mortality risk in incident hemodialysis patients. J Ren Nutr.

[19] Oikonomou KG, Alhaddad A (2016). The diagnostic value of urinalysis in hemodialysis patients with fever, sepsis or suspected urinary tract infection. J Clin Diagn Res.

[20] Narayanaswamy L, Prakasha Murthy RG, Rajappa NG, Patil A, Tharayil AS, Sairaman V (2025). Assessment of intradialytic complications and predisposing factors in chronic kidney disease individuals receiving hemodialysis. Biosci Biotechnol Res Asia.

[21] Dugilo JR, Bakshi F, Abeid M, Somji S (2025). Frequency, predictors and outcomes of intradialytic complications in patients on maintenance haemodialysis in Dar es Salaam: prospective longitudinal study. PLoS One.

[22] Bartaula B, Subedi M, Kumar MM, Shrestha M, Bichha N, Mudbhari B (2019). Spectrum of complications in chronic kidney disease patients undergoing maintenance hemodialysis: an experience of a tertiary care center in Nepal. Saudi J Kidney Dis Transpl.

[23] Sah DK, Heera KC, Shah S, Rai R (2024). Intradialytic complications and its associated factors among chronic kidney disease patients in a tertiary hospital. J Coll Med Sci Nepal.

[24] Patil VD, Yelke BS, Salame R, Madavi T (2024). A study of complications encountered in chronic kidney disease patient undergoing hemodialysis at tertiary health center: a cross-sectional study. Int J Adv Res Med.

[25] Fatima T, Afzal A, Ashraf S (2018). Chronic kidney disease: acute intradialytic complications in chronic kidney disease patients on hemodialysis. Prof Med J.

[26] Hasan S, Kumar H, Prasher PK, Goel R (2017). A study of complications encountered in patients undergoing hemodialysis procedure. Int J Adv Res.

[27] Ibarra-Sifuentes HR, Del Cueto-Aguilera Á, Gallegos-Arguijo DA (2017). Levocarnitine decreases intradialytic hypotension episodes: a randomized controlled trial. Ther Apher Dial.

